# Withdrawal: Mitochondrial pyruvate carrier blockade results in decreased osteoclastogenesis and bone resorption via regulating mitochondrial energy production

**DOI:** 10.1016/j.jbc.2022.102192

**Published:** 2022-07-01

**Authors:** Qian Guo, Hongjian Zhao, Haozhe Cheng, Honglei Kang, Yimin Dong, Renpeng Peng, Meipeng Zhu, Zhong Fang, Feng Li

The manuscript has been withdrawn by the authors due to replicate submissions elsewhere which were initiated concurrent with the submission of a JBC accepted manuscript.

